# Risk factors for anterior communicating artery aneurysm rupture: A protocol for systematic review and meta-analysis

**DOI:** 10.1097/MD.0000000000028088

**Published:** 2021-12-03

**Authors:** Yong Xie, Huan Tian, Bin Xiang, Ding Li, Yu-Zhou Liu, Hua Xiang

**Affiliations:** a Department of Interventional Radiology and Vascular Surgery, the First Affiliated Hospital of Hunan Normal University, Changsha, P. R. China; b Department of Radiology, the Second Affiliated Hospital of Hebei Medical University, Shijiazhuang, P. R. China.

**Keywords:** anterior communicating aneurysm, meta-analysis, risk factors, rupture, systematic review

## Abstract

**Background:**

Although the research on the risk factors of anterior communicating artery (AComA) aneurysm has made great progress, the independent effect of each risk factor on the rupture of AComA aneurysm is controversial among different studies. We will perform a protocol for systematic review and meta-analysis to investigate risk factors for AComA aneurysm rupture and quantify their independent effects.

**Methods:**

A systematic search according to Preferred Reporting Items for Systematic Reviews and Meta-Analysis Protocols guidelines in PubMed, Embase, and the Cochrane Library databases was conducted from inception to August 31, 2021 for published studies concerning risk factors for AComA aneurysm rupture. In the absence of statistical heterogeneity (ie, *P* > .10 and *I*^2^ < 50%), we will use a fixed-effects model to pool the results across sufficient studies. Otherwise, we will present the results employing the random-effects model. Quality assessment of the included studies will be evaluated using the Newcastle–Ottawa Scale. Statistical analyses will be performed using Stata16 (Stata Corporation, College Station, TX, USA) software.

**Results:**

The findings of this study will be submitted to peer-reviewed journals for publication.

**Conclusion:**

This systematic review will provide evidence to determine the risk factors that affect the rupture of the AComA aneurysm and quantify their independent effects.

**Ethics and dissemination:**

Since the proposed study uses pre-published data, ethical approval is not required.

**Review registration number:**

CRD42021284262. (https://www.crd.york.ac.uk/PROSPERO/).

## 1 Introduction

Anterior communicating artery (AComA) aneurysms are more likely to rupture than any other aneurysms in the anterior circulation.^[1–3]^ Rupture of AComA aneurysm accounts for about 30% of non-traumatic subarachnoid hemorrhage.^[4,5]^ The above observations also indicate that the rupture rate of AComA aneurysms is higher than that of other intracranial aneurysms. Due to the improvement of minimally invasive imaging techniques, the detection rate of unruptured aneurysms has increased significantly. Considering the high mortality and morbidity caused by rupture, it is important to determine the risk factors that trigger AComA aneurysm rupture, which is conducive to the risk stratification of AComA aneurysm.^[6]^ In the international study of unruptured intracranial aneurysms, it is reported that the size and location of unruptured aneurysms are the most important determinants of rupture.^[7]^ Other subsequent studies have shown that additional parameters such as smaller aneurysm neck diameter, irregular aneurysm shape, and higher aspect ratio, size ratio, flow angle, and current smoking may also affect the rupture of AComA aneurysm.^[8–11]^

However, it is not clear which morphological parameter or hemodynamic parameter is more decisive. Identifying the factors that influence the risk of rupture in patients with unruptured AComA aneurysms will guide the determination of clinical decisions to improve the patient's prognosis. To gain more insight into this topic, we, therefore, will perform a protocol for systematic review and meta-analysis aiming to determine the risk factors that affect the rupture of the AComA aneurysm and quantify their independent effects.

## 2 Methods

### 2.1 Study protocol registration

This meta-analysis will be carried out using the protocol designated by the Cochrane collaboration^[12]^ and reported based on the items of Preferred Reporting Items for Systematic Reviews and Meta-Analysis Protocols.^[13]^ The review protocol has been registered on the PROSPERO (https://www.crd.york.ac.uk/PROSPERO/).

### 2.2 Search strategy

Two investigators will independently search the literature published in Embase, the Cochrane Library, and PubMed from inception to August 31, 2021. The main search terms used for the search are “aneurysm,” “anterior communicating aneurysm,” “AComA aneurysm,” “AComA,” “anterior cerebral artery aneurysm,” “rupture,” “ruptures,” “associated factors,” “risk factors.” There is no language restriction for this search, and the reference list of all selected articles will be filtered to identify other studies.

### 2.3 Selection criteria

#### 2.3.1 The inclusion criteria:

(a) Patients: patients with AComA aneurysm (diagnosed by computed tomography or magnetic resonance imaging or digital subtraction angiography).
(b) Study type: cohort, case–control, or cross-sectional studies.

#### 2.3.2 The exclusion criteria

(a) Articles with missing data or articles containing only abstract or duplicate data.
(b) Letters, meta-analyses, reviews, comments, animal trials, or meeting articles.

### 2.4 Data selection and extraction

**Figure 1:**
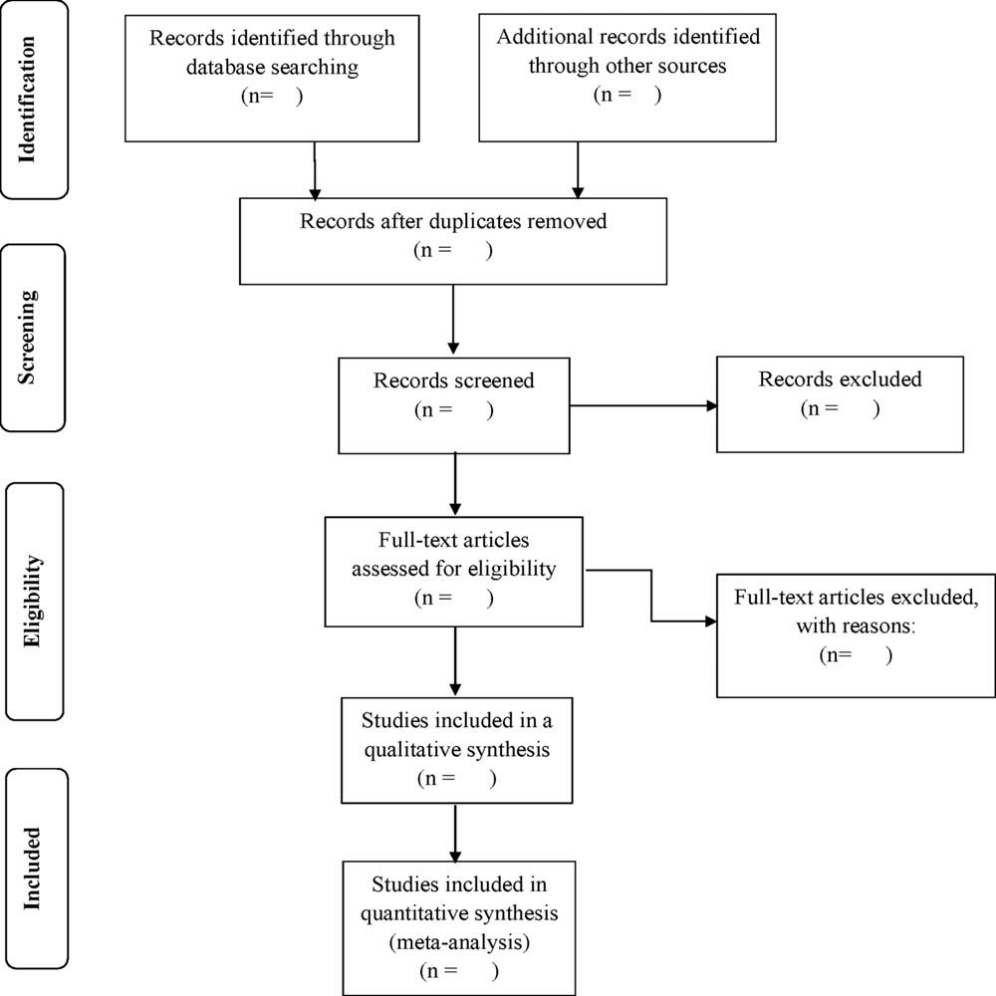
PRISMA flow diagram of the study selection process. PRISMA-P = Preferred Reporting Items for Systematic Reviews and Meta-Analysis Protocols.

First, 2 investigators will identify published pieces of literature in our initial extensive search, and then use Endnote X9 software to conduct a preliminary assessment of the title and abstract of each document in the database. After carefully reading titles and abstracts, appropriate literature will be selected for possible inclusion. Then, after a review of full text, eligible cohort, case–control or cross-sectional studies will be found to meet the inclusion criteria. Figure 1 shows the flow diagram related to the selection of study articles. When identifying overlapping observational researches, we will only recruit the most comprehensive and latest report for final analysis. Two authors will independently extract variables using a prespecified data-collection sheet. For the final included studies, we will extract the following baseline data: first author's name, publication year and publication journal, study design, country, baseline characteristics of the patient (eg, sex, age, and concomitant disease states such as hypertension); aneurysm morphological parameters (eg, size, location, shape, aspect ratio, aneurysm neck width, etc), and information regarding the associated factors. Any disagreements are discussed and resolved by the third reviewer.

### 2.5 Quality assessment

We use the Newcastle–Ottawa Scale, which is used to assess the quality of observational studies, to assess the quality of included studies. This tool includes 3 items: selection, comparability, and outcomes.^[14]^ Articles with a score of 6-9 were considered high quality, whereas research with a rating of <6 stars is considered low-quality research. Two reviewers will independently assess the quality of included studies. Any disagreements are discussed and resolved by the third reviewer.

### 2.6 Statistical analysis

For effect sizes of risk factors will be pooled by odds ratio or risk ratio with 95% confidence intervals. Statistical heterogeneity between studies will be examined using Cochran *Q* test, while Higgin *I*^*2*^ statistic is calculated to quantify the heterogeneity of the included trials. In the absence of statistical heterogeneity (ie, *P* > .10 and *I*^2^ < 50%), we will use a fixed-effects model to pool the results across sufficient studies. Otherwise, we will present the results employing the random-effects model. Potential publication bias will be assessed by funnel plots and the Egger test. If necessary, we will perform a sensitivity analysis to explore the sources of heterogeneity and the results’ stability. All *P* values (two-sided) with *P* < .05 are considered to be statistically different. We use statistical software Stata16 (Stata Corporation, College Station, TX, USA) for all statistical analyses.

## 3 Discussion

AComA is the most common site of aneurysm rupture, with an incidence of about 30%.^[15]^ Although the incidence of subarachnoid hemorrhage seems to decrease due to the intervention of its risk factors,^[16]^ aneurysm rupture is still an important factor leading to high mortality and morbidity.^[17]^ However, it is not clear which morphological parameter or hemodynamic parameter is more decisive. Furthermore, existing researches on this topic usually have a small sample size, various imaging techniques, and most are observational trials, therefore, it is necessary to develop this systematic review and meta-analysis to combine the available clinical evidence. We hope this study will provide more comprehensive, and reliable evidence for clinical decision-making and future research. Future prospective studies involving a larger patient population are necessary, which may provide us with stronger evidence to help us more fully answer the challenges and problems leftover from this topic.

## Author contributions

**Conceptualization:** Yong Xie, Huan Tian.

**Data curation:** Yong Xie, Huan Tian, Yu-Zhou Liu, Ding Li.

**Funding acquisition:** Hua Xiang.

**Methodology:** Yong Xie, Huan Tian, Bin Xiang, Ding Li.

**Supervision:** Bin Xiang, Hua Xiang.

**Writing – original draft:** Yong Xie, Huan Tian.

**Writing – review & editing:** Yong Xie, Huan Tian, Bin Xiang, Hua Xiang.
